# Draft Genome Sequence of Multidrug-Resistant *Cellulosimicrobium* sp. Strain KWT-B, Isolated from Feces of *Hirundo rustica*

**DOI:** 10.1128/genomeA.00641-17

**Published:** 2017-07-13

**Authors:** Takehiko Kenzaka, Yuina Ishimoto, Katsuji Tani

**Affiliations:** Environmental Science and Microbiology, Faculty of Pharmacy, Osaka Ohtani University, Nishikiori-kita, Tondabayashi, Japan

## Abstract

Migratory birds have been postulated as potential spreaders of antibiotic resistance. Multidrug-resistant *Cellulosimicrobium* sp. strain KWT-B was isolated from the feces of *Hirundo rustica*. A draft genome sequence indicated that the strain harbors multidrug-resistant transporters, multidrug efflux pumps, a vancomycin-resistant protein, and metallo-beta-lactamases.

## GENOME ANNOUNCEMENT

Many avian species have been found to carry antibiotic-resistant bacteria and resistance genes (1). Because of their ability to migrate to long distances in short periods, migratory birds are a possible source of antibiotic-resistant bacteria that colonize and/or infect human beings (2). *Hirundo rustica* (barn swallow) is the most widespread species of swallows in the world. Their global population is estimated to be approximately >190,000,000 individuals (3). In Japan, *H. rustica* populations migrate from Southeast Asia to the whole region during the spring and breed in and migrate back to Southeast Asia during autumn (4). This migratory population is estimated to be some hundreds of thousands of individuals. However, the incidence and type of antibiotic-resistant bacteria that are associated with migratory birds in East Asia remain unclear.

Multidrug-resistant *Cellulosimicrobium* sp. strain KWT-B was isolated on medium containing meropenem, ciprofloxacin, and amikacin from the feces of *H. rustica*, and its draft genome sequence is presented here. 16S rRNA sequence analysis revealed that strain KWT-B had 99% similarity to *Cellulosimicrobium* sp. strain PONa. Members of the genus* Cellulosimicrobium* are characterized as Gram-positive, rod-shaped, nonmotile chemoorganotrophs (5). They have been found in the soil, marine sponges, hot springs, Antarctic snow, compost, and agricultural soil, and *Cellulosimicrobium* bacteria can cause infections in humans (6–12).

The draft genome was sequenced by 300-bp paired-end sequencing on an Illumina Miseq sequencing system (Fasmac Co. Ltd., Atsugi, Kanagawa, Japan). High-quality sequence reads (3,394,741 pairs) were assembled *de novo* using SPAdes version 3.6.0 (13). The final assembly of the genome produced 4,412,091 bp in 16 contigs, with an *N*_50_ of 821,157 bp and a G+C content of 74.6%. The assembled contigs were functionally annotated using the RAST server (14). The genomes contained 3,784 putative coding sequences (CDSs). One copy each of 23S rRNA and 16S rRNA and four copies of 5S rRNA in the draft genome were revealed. The strain KWT-B lacked detectable plasmids, but it had multidrug resistance. MIC values were >256 mg liter^-1^ for amikacin, 12 mg liter^-1^ for ciprofloxacin, 12 mg liter^-1^ for imipenem, 24 mg liter^-1^ for colistin, and 0.19 mg liter^-1^ for vancomycin.

The genome of strain KWT-B encodes some multidrug-resistant proteins, resistance-nodulation-division (RND) family efflux transporters, Na^+^-driven multidrug efflux pump proteins, the drug resistance transporter EmrB/QacA subfamily, two drug resistance transporter Bcr/CflA subfamilies, the multidrug efflux pump subunit AcrB, the arabinose efflux permease major facilitator superfamily (MFS), vancomycin-resistant protein W, and four metallo-hydrolase-like–metallo-β-lactamase (MBL)-fold superfamilies. They are conserved in previously sequenced *Cellulosimicrobium* species (15). These results suggest that *H. rustica* can spread multidrug-resistant* Cellulosimicrobium* spp. through migration between Japan and Southeast Asia and that the bacteria can be transmitted from birds to humans and vice versa. The genome of *Cellulosimicrobium* sp. KWT-B will facilitate understanding of the ecology and global distribution of *Cellulosimicrobium* spp. via migratory birds. Studies on *Cellulosimicrobium* associated with swallows may help improve the understanding of the dissemination of antibiotic resistance in the environment.

### Accession number(s).

The draft genome sequence of *Cellulosimicrobium* sp. KWT-B has been deposited in the DDBJ/EMBL/GenBank with the accession number NEDO00000000.
